# Synthesis and Characterisation of Copper(II) Complexes with Tridentate NNO Functionalized Ligand: Density Function Theory Study, DNA Binding Mechanism, Optical Properties, and Biological Application

**DOI:** 10.1155/2014/104046

**Published:** 2014-10-16

**Authors:** Madhumita Hazra, Tanushree Dolai, Akhil Pandey, Subrata Kumar Dey, Animesh Patra

**Affiliations:** ^1^Postgraduate Department of Chemistry, Midnapore College, Midnapore 721101, India; ^2^Department of Chemistry, Sidho-Kanho-Birsha University, Purulia, West Bengal 723101, India; ^3^Department of Microbiology, Midnapore College, Midnapore 721101, India

## Abstract

The photo physical properties of two mononuclear pentacoordinated copper(II) complexes formulated as [Cu(L)(Cl)(H_2_O)] (**1**) and [Cu(L)(Br)(H_2_O)] (**2**) **HL** = (1-[(3-methyl-pyridine-2-ylimino)-methyl]-naphthalen-2-ol) were synthesized and characterized by elemental, physicochemical, and spectroscopic methods. The density function theory calculations are used to investigate the electronic structures and the electronic properties of ligand and complex. The interactions of copper(II) complexes towards calf thymus DNA were examined with the help of absorption, viscosity, and fluorescence spectroscopic techniques at pH 7.40. All spectroscopy's result indicates that complexes show good binding activity to calf thymus DNA through groove binding. The optical absorption and fluorescence emission properties of microwires were characterized by fluorescence microscope. From a spectroscopic viewpoint, all compounds strongly emit green light in the solid state. The microscopy investigation suggested that microwires exhibited optical waveguide behaviour which are applicable as fluorescent nanomaterials and can be used as building blocks for miniaturized photonic devices. Antibacterial study reveals that complexes are better antimicrobial agents than free Schiff base due to bacterial cell penetration by chelation. Moreover, the antioxidant study of the ligand and complexes is evaluated by using 1,1-diphenyl-2-picrylhydrazyl (DPPH) free-radical assays, which demonstrate that the complexes are of higher antioxidant activity than free ligand.

## 1. Introduction

Copper(II) complexes play an important role in the active sites of a large number of metalloproteins in biological systems and potential application for numerous catalytic processes in living organisms that involve electron transfer reactions or activation of some antitumor substances [1]. These processes are also involved inbioinorganic [2] and medicinal chemistry [3]. In fact copper(II) chelates have been found to interact with biological systems and to exhibit antineoplastic activity [4–6] and antibacterial, antifungal [7, 8], and anticancer activity [9]. Some copper(II) N,S,O/N,N-donor chelators are good anticancer agents due to strong binding ability with DNA base pair [10]. Pyridines are common but vital heterocyclic compounds in organic synthesis, especially as agrochemicals and synthetic intermediates. For example, pyridine derivatives, such as (aromatic) alkoxyl pyridine compounds, amidopyridine, and its derivatives, substituted fused pyridine compounds, have already been widely applied in the fields of agrochemical products [11]. Moreover, pyridine derivatives play a unique role in anthelminthic, acaricide, bacteriocide, and phytocide [12]. For these biological effects we choose the pyridine derivative ligand as a starting material.

DNA biopolymer plays essential role in the growth, development, and heritage transmission of living species not only of humans and animals but also of the vegetal ones. This is one of the most important substances in biological system, whose base pairs carrying the genetic information related not only to the normal life activity but also to the abnormal activities such as carcinogenesis. Compounds having ability to bind and cleave double stranded DNA under physiological conditions are of importance for their utility as diagnostic agents in medicinal applications and for genomic research. DNA base pairs and amino sugar moiety are involved mainly in intercalative or groove binding interactions. In this respect the design of functional materials has received considerable attention due to their propensity to take part also in potential applications in DNA molecule probes [13]. Fluorescence spectroscopy measurement also helps in studying the dynamic interactions and apparition of macromolecules and macromolecular complexes. The relevance of fluorescence techniques to a range of bioanalytical, biophysical assays is based on the use of different fluorescence probes that can interact with macromolecules and with nucleic acids (DNA and RNA). The applications of DNA in photonics and optoelectronics have attracted intensive attentions during recent years [14] because the DNA-lipid complex has thermal and optical stability [15].

**Scheme 1:**
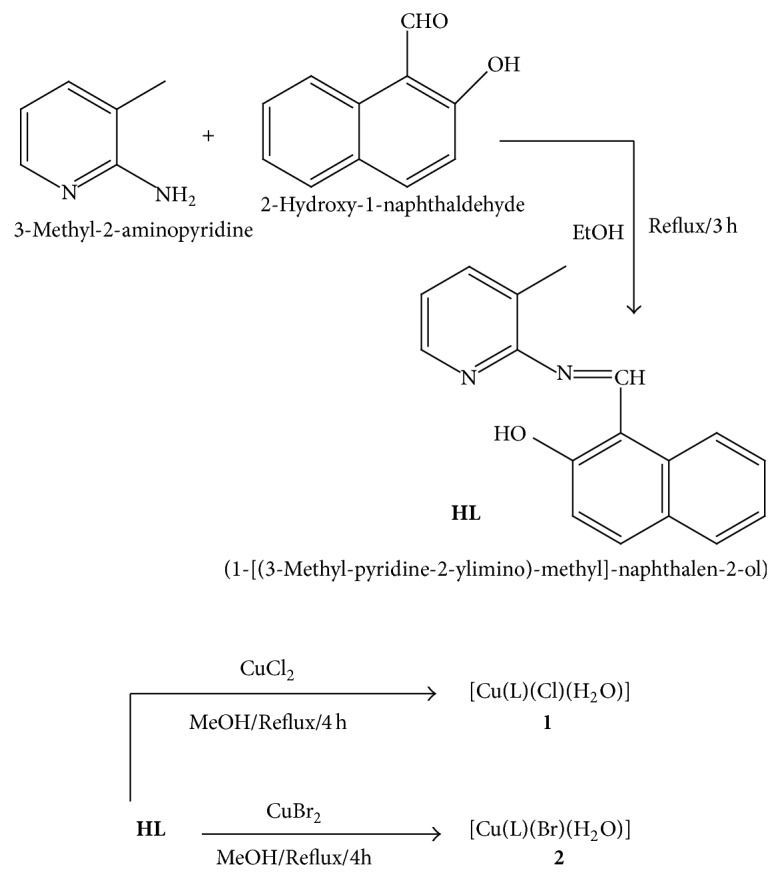
Synthetic procedure of the ligand and its copper(II) complexes.

Herein we report an account of fluorescent mononuclear copper(II) complexes obtained with tridentate NNO-donor ligand (1-[(3-methyl-pyridine-2-ylimino)-methyl]-naphthalen-2-ol) (**HL**) (vide Scheme 1). The electron transfer mechanism of copper(II) complexes is investigated by cyclic voltammetry. The density function theory calculations are used to examine the electronic properties of these complexes. The DNA binding study of the copper(II) complexes has been performed spectroscopically. Here, we report the synthesis of new copper complexes nanowires with DNA and explain fluorescence emission properties. From a spectroscopic study, copper(II) complexes strongly emit green light in the solid state. DNA optical microwire devices are expected to be used as optical biosensors. The antioxidant study of the ligand and complexes is evaluated by using DPPH free-radical assays. Antibacterial activity of the Schiff base and its copper complexes has also been studied by agar disc diffusion method against some species of pathogenic bacteria (*Escherichia coli*,* Vibrio cholerae*,* Streptococcus pneumonia*,* and Bacillus cereus*).

## 2. Materials and Physical Measurements

All chemicals and reagents were obtained from commercial sources and used as received, unless otherwise stated. Solvents were distilled from an appropriate drying agent. The organic moieties were synthesized following the procedure. The elemental (C, H, N) analyses were performed on a Perkin Elmer model 2400 elemental analyzer. Copper analysis was carried out by Varian atomic absorption spectrophotometer (AAS) model-AA55B, GTA using graphite furnace. Electronic absorption spectra were recorded on a SHIMADZU UV-1800 spectrophotometer. The fluorescence spectra of EB bound to DNA were obtained in the fluorimeter (Hitachi-2000). Electron spray ionization (ESI) mass spectra were recorded on a Qtof Micro YA263 mass spectrometer. IR spectra (KBr discs, 4000–400 cm^−1^) were recorded using a Perkin Elmer FTIR model RX1 spectrometer. The room temperature magnetic susceptibility measurements were performed by using a vibrating sample magnetometer PAR 155 model. Molar conductance (Λ_M_) was measured in a systronics conductivity meter 304 model using ~10^−3^ mol L^−1^ solutions in DMF solvent. Optical microscopy images were taken using an NIKON ECLIPSE LV100POL upright microscope equipped with a 12 V-50 W halogen lamp. The samples for optical microscopic study were prepared by placing a drop of colloidal solution onto a clean glass slide. Electrochemical measurements were performed using computer-controlled CH-Instruments (Model No. CHI620D). All measurements were carried out under nitrogen environment at 298 K with reference to SCE electrode in dimethyl formamide using [*n*-Bu_4_N]ClO_4_ as supporting electrolyte. Stock solutions of complex-**1** and complex-**2** were prepared in DMF because of their lower solubility in water.

### 2.1. Preparation of the Ligand (**HL**)

The synthesis of ligand** HL** was prepared by modifying the reported procedure [16]. An ethanolic solution of 2-hydroxy-naphthaldehyde (0.86 g, 5.0 mmol) was added to 3-methyl-2-aminopyridine (0.64 g, 5.0 mmol) in ethanol. Then this mixture was allowed to stir at room temperature for 2 h and then it was refluxed for 3 h. The mixture was cooled to room temperature and kept over one night to get the precipitate of the solid orange ligand. The precipitate was filtered by using vacuum pump and washed several times using ethanol to remove any unreacted materials; then product was collected by recrystallization from ethanol and dried in vacuum desiccators. Finally the product was characterized by IR, ^1^H NMR, and ^13^C NMR spectroscopy.

C_17_H_14_N_2_O: anal. Found: C, 77.86; H, 5.34; N, 10.68; Calc.: C, 77.82; H, 5.28; N, 10.44, m.p. 186 ± 1°C; IR (KBr, cm^−1^):* v*_O–H_, 3448,* v*_C=N_, 1472,* v*_CH=N_, 1623; ^1^H NMR (*δ*, ppm in CDCl_3_ + CCl_4_): 15.686 (d, 1H_a_); 9.976 (d, 1H_b_); 8.30 (d, 1H_c_); 6.89 (d, 1H_d_); 8.15–7.04 (m, 9H); 2.498 (s, 1H_e_); ^13^C NMR: 149.08 (C-9), 146.31 (C-1), 139.45 (C-7), 129.32–119.31 (Ar-C), 17.00 (C-6); yield: 90%.

### 2.2. Preparation of [Cu(L)(Cl)(H_2_O)] **(1)** and [Cu(L) (Br)(H_2_O)] **(2)**

To prepare the copper(II) complexes (**1 **and** 2**) a common procedure (Scheme 1) was followed as described below, using copper(II) chloride for complex (**1**), copper(II) bromide (**2**), and the organic ligand (**HL**) in equimolar (1 : 1) ratio. A methanolic solution of** HL** (1.0 mmol) was mixed with 1.0 mmol of copper(II) chloride (**1**) and copper(II) bromide (**2**) with stirring condition and the mixture was refluxed for 4 h. The solid product was collected by filtration and washing with cold methanol and water then dried in vacuo. The pure crystallized product was obtained from methanol.

[Cu(L)(Cl)(H_2_O)] (**1**): yield 80–85%; C_17_H_15_N_2_O_2_ CuCl

Complex-**1**: C_17_H_15_N_2_O_2_CuCl: Anal. Found; C, 53.96; H, 3.96; N, 7.40; Cu, 16.81; Calc: C, 53.84; H, 3.92; N, 7.34; Cu, 16.72. IR (cm^−1^):* v*_CH=N_, 1618;* v*_C=N_, 1470,* v*_O–H_, 3438. m.p. 232 ± 1°C. ESI MS (*m*/*z*): M^+^ 378, [M+2]^+^ 380. Magnetic moment (*μ*, B.M.): 1.74. Conductivity (Λo, S cm^−1^) in DMF: 6.32.

[Cu(L)(Br)(H_2_O)] (**2**): yield 75–80%; C_17_H_15_N_2_O_2_ CuBr

Complex-**2**: C_17_H_15_N_2_O_2_CuBr: Anal. Found; C, 48.29; H, 3.55; N, 6.62; Cu, 15.03; Calc: C, 48.18; H, 3.48; N, 6.54; Cu, 14.82. IR (cm^−1^):* v*_CH=N_, 1620;* v*_C=N_, 1468,* v*_O–H_, 3440. m.p. 243 ± 1°C. ESI MS (*m*/*z*) M^+^ 422, [M+2]^+^ 424. Magnetic moment (*μ*, B.M.): 1.72. Conductivity (Λo, S cm^−1^) in DMF: 6.30.

### 2.3. Theoretical Methodology

All molecular calculations were performed in the gas phase using density functional theory (DFT) with B3 [17] LYP [18] with [1] exchange correlation functional. The basis set 6-31G (d, p) was used for all atoms [19]. All calculations were carried out using the GAUSSIAN 09 program package with the aid of the Gauss View visualization program [20].

### 2.4. DNA Binding Experiments

The DNA binding experiments were done by a Tris-HCl buffer (pH 7.4) with copper complexes in DMF solvent. The DNA concentration per nucleotide was determined by absorption spectroscopy using the molar absorption coefficient (6600 (mol L^−1^)^−1 ^cm^−1^) at 260 nm. A solution of DNA in the buffer gave a ratio of UV absorbance at 260 and 280 nm of about 1.8-1.9, indicating that the DNA was sufficiently free of protein [21]. Absorption spectral titration experiment was performed by keeping the constant concentration of the copper(II) complex and varying the CT-DNA concentration. After addition of DNA to the copper complex, the resulting solution was allowed to equilibrate at 25°C for 30 min, after which absorption spectra were noted.

Ethidium bromide displays very weak fluorescence in aqueous solution. However, in the presence of DNA, it exhibits intense fluorescence because of the intercalation to base pairs in DNA. In the ethidium bromide (EB) fluorescence displacement experiment, 5 *μ*L of the EB Tris-HCl solution (1 mmol L^−1^) was added to 1 mL of DNA solution [22], stored in the dark for 2 h. Then the solution of the copper(II) complex was titrated into the DNA/EB mixture and diluted in Tris-HCl buffer to 5 mL to get the solution with the appropriate complex/CT-DNA mole ratio. Prior to measurements, the mixture was shaken up and incubated at room temperature for 30 min. Fluorescence measurements were performed at an excitation wavelength of 522 nm, and the emitted fluorescence was analyzed at 610 nm.

### 2.5. Determination of Viscosity

Viscosity experiments were conducted on an Ostwald's viscometer. The concentration of the copper(II) complexes (**1** and** 2**) varying from 0.5 to 4.0 × 10^−6^ M and each complex was introduced into a DNA solution (5.25 × 10^−6^ M) present in the viscometer. Each sample was measured two times and average flow time was calculated. The values of relative viscosities of DNA in the absence and presence of the complexes are plotted against the ratio of the concentration of complex and CT-DNA [23].

### 2.6. Antimicrobial Screening

The antibacterial activity of the tested samples was determined using a modified agar disc diffusion method [24]. The activities were done at 100 and 200 *μ*g/mL concentrations of ligand and its copper(II) complexes in DMF solvent by using three pathogenic Gram negative bacteria (*Escherichia coli*,* Vibrio cholerae*,* and Streptococcus pneumoniae*) and one Gram positive pathogenic bacteria (*Bacillus cereus*). The solution of ligand and its copper(II) complexes were added to the agar plates and incubation of the plates was done at 37°C for 24 hours. At the end of the period, the diameter of the inhibition zones was calculated in millimetres [25].

### 2.7. DPPH Radical Scavenging Activity

Antioxidant activity of the synthesized compounds was estimated by 1,1-diphenyl-2-picrylhydrazyl (DPPH) radical scavenging effect. The 0.1 mL of different concentrations (25 to 150 *μ*g/mL) of sample in methanol was added to 4 mL of a 1.46 × 10^−5^ M DPPH solution, and then solution was left to stand at room temperature in the dark. After 30 min of incubation, the absorbance of the solution was measured at 520 nm [26].

### 2.8. Synthesis and Characterization

The organic ligand (**HL** = (1-[(3-methyl-pyridine-2-ylimino)-methyl]-naphthalen-2-ol)) was synthesized by the reaction of the respective 3-methyl-2-aminopyridine (5 mmol) and then 5.0 mmol of 2-hydroxy-1-naphthaldehyde in presence of ethanol. The complexes were obtained in good yield from the reaction of the copper chloride (**1**) and copper bromide (**2**) with equimolar amount of organic moiety** HL** in the methanol medium. In these complexes the organic molecule** HL** acts as tridentate ligand through NNO donor centres. The complexes conductivity measurement in DMF suggests that complexes exist in solution as nonelectrolytes [27]. These complexes are air-stable, coloured solids, partly soluble in ethanol and methanol, and soluble in acetonitrile, DMSO, and DMF. All copper(II) complexes are nonhygroscopic and monomeric in nature. At room temperature the magnetic moments (*μ*) of these complexes are 1.74 and 1.72 B.M. Satisfactory analytical results were obtained for all the complexes, exhibiting paramagnetic character comparable to mononuclear copper(II) complexes of tridentate Schiff bases [28]. From conductivity, UV-Vis spectra and magnetic moment measurement indicate all complexes are distorted trigonal bipyramidal geometry [29].

### 2.9. Infrared and Electronic Spectral Studies

The IR spectrum of the ligand has several bands appearing at 3448, 1472, and 1623 cm^−1^ due to phenolic O–H group, pyridine C=N, and imine CH=N stretching vibrations in the solid state (see Figure S1 in Supplementary Material available online at http://dx.doi.org/10.1155/2014/104046). The hydroxyl hydrogen of ligand is replaced by a metal in metal complexes. In complexation the bands are shifted to lower frequency at 409–411 and 514–518 cm^−1^ which are attributed to the existence of Cu–O and Cu–N bond with copper(II) ion. These vibrations confirmed the involvement of nitrogen and oxygen atom in chelation with metal ion. Hence, a broad band in the range of 3,438–3,440 cm^−1^ indicates the presence of a water molecule in complex-**1** and complex-**2**. All the IR data suggest that the metal ions are coordinated to the Schiff base through the phenolic oxygen, imino-nitrogen, and pyridine nitrogen and with one water molecule.

The proton NMR spectra of the free ligand have been recorded in CDCl_3_ at room temperature using CCl_4_ as an internal standard (Figure S2). The ligand exhibits hydroxyl proton (H_a_) appearing at *δ* 15.68 ppm, the aromatic pyridine proton (H_b_) appearing at *δ* 9.97 ppm, H_c_ appearing at *δ* 8.30 ppm, H_d_ appearing at *δ* 6.89 ppm, methyl proton (H_e_) appearing at *δ* 2.49 ppm, and aromatic and heteroaromatic proton signals appearing at *δ* 7.04–8.15 ppm. The chemical shift of hydroxyl proton is very high (15.68 ppm) indicating intramolecular hydrogen bond (inset Figure S2). ^13^C NMR spectra (Figure S3) showed similar diagnostic features for the free ligand. Hydroxyl carbon (C-9) was found at 149.08 ppm, pyridine carbon (C-1) at 146.31 ppm, and imine carbon (C-7) at 139.45 ppm, and the methyl carbon (C-6) signal was found at 17.0 ppm and aromatic carbons were found at 119.3–129.3 ppm. NMR spectra of the free ligand support the conclusions derived from the IR spectra.

The electronic spectra of all complexes were recorded in DMF at room temperature. The electronic spectral data of the Schiff base and their complexes are given in Table 1. All the spectra of complexes show lower bands than 400 nm due to *π* → *π* ^*^ and *n* → *π* ^*^ transitions for the aromatic ring, and again absorption bands at 436.0 nm and 456 nm are due to intraligand charge transfer transitions. An intense band at 262 nm is assigned to *π* → *π* ^*^ intraligand transition [30] along with the less intense bands at 319 and 365 nm corresponding to the ligand to metal charge transfer transition. The copper(II) complex-**1** and complex-**2** show a d–d broad and a weak band centered at 664 and 672 nm which is attributed to ^2^B_1g_ → ^2^A_1g_ transition [31]. This electronic spectrum is compared with five coordinate complexes consistent with the degree of distortion from the TBP geometry [29, 32].

### 2.10. Electron Sprays Ionization Mass Spectra (ESI MS)

The mass spectra of complexes (Figures S4 and S5) support their projected formulation. It reveals the molecular ion peak *m*/*z* at 262.16, consistent with the molecular weight of the ligand, whereas its copper complexes (**1** and** 2**) show a weak molecular ion peak at *m*/*z* 378.14 and 422.6 due to the higher instability. A weak peak at *m*/*z* 380 and 424 corresponds to the [M+2]^+^ peak possibly due to the presence of isotopic chlorine and bromine in the copper complexes of** 1** and** 2**, respectively [33, 34]. Other peaks were observed at *m*/*z* 361, 324, 248, and 161 which corresponds to different fragments that support the structure of the copper complexes.

### 2.11. Electrochemistry

The redox properties of the Cu(II) complexes were examined by cyclic voltammetry using a Pt-disk working electrode and a Pt-wire auxiliary electrode in dry dimethylformamide using [*n*-Bu_4_N]ClO_4_ (0.1 M) as the supporting electrolyte. The cyclic voltammetric data are given in Table 1. The cyclic voltammograms exhibit quasi-reversible electron transfer process with a reduction peak at *E*_pc_ = −0.779 V  and −0.712 V with a corresponding oxidation peak at *E*_pa_ = −0.602 V and −0.597 V for complex-**1** and complex-**2**, respectively, at a scan rate interval 50–400 mV s^−1^. The *E*_1/2_ values for these Cu(II)/Cu(I) redox couples were in the range of −0.690 to −0.654 V versus Ag/AgCl and the ratio of cathodic to anodic peak height was less than one. The most significant feature of the Cu(II) complexes are the Cu(II)/Cu(I) couple [35]. The ratio between the cathodic peak current and the square root of the scan rate (*I*_pc_/*v* ^1/2^) is approximately constant. From this cyclic voltammetry data it can be deduced that the redox couples are related to a quasi-reversible one-electron transfer process.

### 2.12. Emission Activity

**Figure 1:**
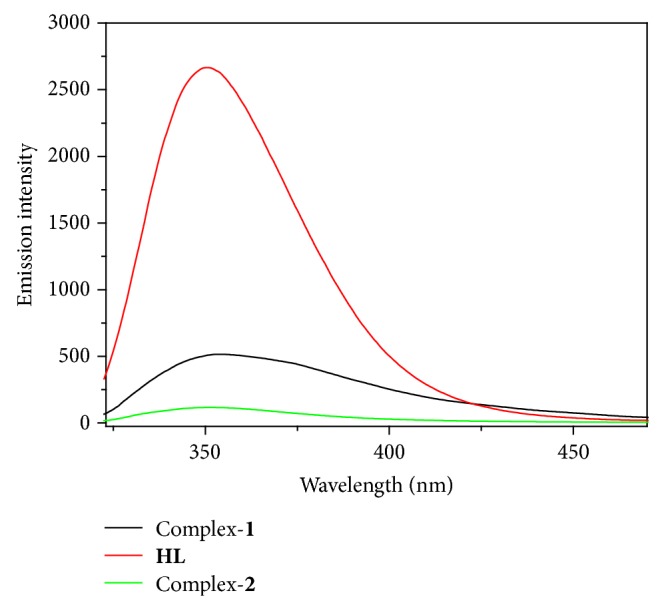
Fluorescence emission properties of the free ligand (**HL**) and its copper(II) complexes.

The emission property of the ligand** HL** and its copper(II) complexes was recorded at room temperature (298 K) in 1 × 10^−6^ (M) DMF solution given in Figure 1. In the absence of metal ions the fluorescence of the ligand is probably quenched by the occurrence of a photo induced electron transfer (PET) process due to the presence of lone pair of electrons in the ligand [36]. It is evident that the fluorescence emission intensity of the ligand decreases dramatically depending on the complex formation with the metal ions. These coordination complexes make the energy transfer from the excited state of the ligand to the metal ions causing decreases of the fluorescence intensity. For this reason the intensity of complex-**1** and complex-**2** is decreased. The ligand shows higher fluorescence intensity at 352 nm while complex-**2** is fluorescence silent when both are excited at 300 nm in DMF solution.

### 2.13. Electronic Structure

**Figure 2:**
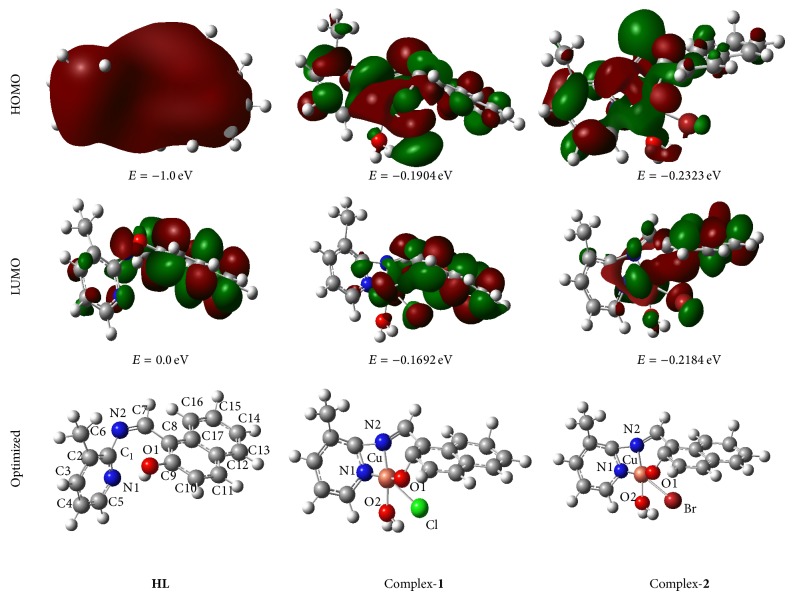
The HOMO and LUMO orbitals of** HL** and copper(II) complex-**1** and complex-**2** obtained from DFT.

Full geometry optimization of** HL** and copper(II) complexes (**1** and** 2**) was carried out using density functional theory (DFT) at the B3LYP level in their ground state shown in Figure 2. The frontier orbitals of HOMO and LUMO of** HL**,** 1**, and** 2** are also given in Figure 2. The selected bond distances and bond angles are reported in Table S1. Thus it is apparent that electron density of HOMO in** HL** is largely localized on both pyridine and naphthalene ring. HOMO of copper(II) complexes have largely localized on pyridine ring and partly on naphthalene but in LUMO electrons are largely localized on naphthalene ring. The HOMO-LUMO energy gap in the ground state of complex-**1** and complex-**2** has been predicted to be 0.0212 and 0.0139 eV, respectively, and is not influenced by excitation. From this optimized structure, the bond length of Cu–Cl is 2.16 Å and of Cu–Br is 2.29 Å; this suggests that larger size of bromine atom forms weaker overlap with copper atom but other bond lengths are comparable. The N1–C1–N2 bond angle of** HL** is 119.30° but on complex formation the bond angle decreases to 85.36° and 86.13° for** 1** and** 2**, respectively.

### 2.14. DNA Binding Studies

The binding interaction of the copper(II) complexes with calf thymus DNA has been investigated with the help of spectroscopic, viscosity measurements, and electrochemical study.

### 2.15. Electronic Absorption Study

**Figure 3:**
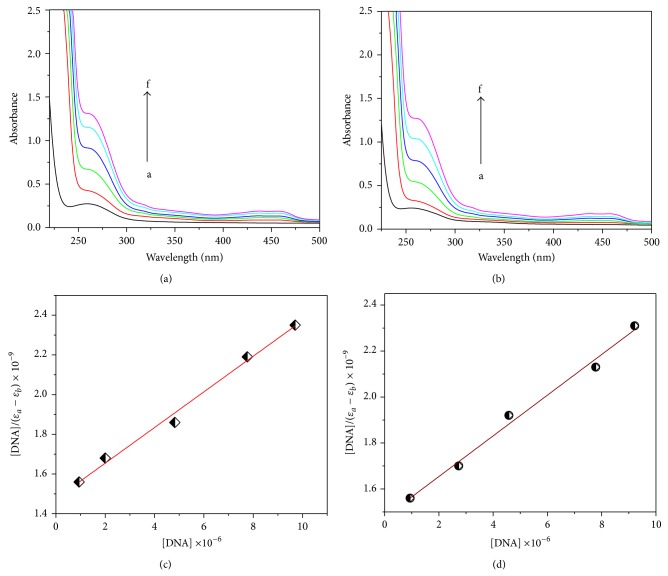
Electronic spectral titration (a, b) of complex-**1** (a) and complex-**2** (b) with CT-DNA at 266 nm in Tris-HCl buffer; [complex] = 2.34 × 10^−5^; [DNA]:* a* 0.0,* b* 1.22 × 10^−6^,* c* 2.44 × 10^−6^,* d* 3.66 × 10^−6^,* e* 4.88 × 10^−6^,* f* 6.10 × 10^−6^ mol L^−1^. The arrow denotes the gradual increase of DNA concentration. Plot of [DNA]/(*ε*_*a*_ − *ε*_*f*_) versus [DNA] for the absorption titration of CT-DNA with the copper(II) complex-**1** (c) and complex-**2** (d) in Tris-HCl buffer at the (c, d).

In general, the hyperchromism and hypochromism were regarded as spectral features for DNA double-helix structural change when DNA reacted with other molecules. The hyperchromism originates from the breakage of the DNA duplex secondary structure; the hypochromism originates from the stabilization of the DNA duplex by either the intercalation binding mode or the electrostatic effect of small molecules. It is reported that if the aromatic ring of the molecule closely matches with the helical turn of the CT-DNA groove, the aromatic rings of the ligand interact with DNA in Tris-HCl buffer through the formation of the van der Waals contacts or hydrogen bonds in the DNA grooves. The binding of the copper(II) complex to the CT-DNA helix is examined by an increase of the absorption band (c.a. 264 nm) of copper(II) complex. This increasing absorbance indicates that there is the involvement of strong interactions between complex and the base pairs of DNA [37]. The absorption spectra of the copper(II) complexes in the absence and presence of CT-DNA are shown in Figure 3. A hyperchromism was also observed for a copper(II) complex with a ligand bearing *-*OH group. The extent of the hyperchromism in the charge transfer band is generally consistent with the strength of interaction [38]. As DNA double helix possesses many hydrogen bonding sites which are accessible both in the minor and in the major grooves, it is likely that the –OH group of the ternary complex forms hydrogen bonds with DNA, which may contribute to the hyperchromism observed in absorption spectra. The increasing absorbance indicates there is groove binding modes.

In order to further illustrate the binding strength of the copper(II) complex with CT-DNA, the intrinsic binding constant *K*_*b*_ was determined from the spectral titration data using the following equation [39]:

(1) $$\begin{array}{c} \frac{\left[\text{DNA}\right]}{\left(\varepsilon_{a}-\varepsilon_{f}\right)}\;=\frac{\left[\text{DNA}\right]}{\left(\varepsilon_{a}-\varepsilon_{f}\right)}+\frac{1}{\left[K_{b}\left(\varepsilon_{b}-\varepsilon_{f}\right)\right]}, \end{array}$$

where [DNA] is the concentration of DNA, *ε*_*f*_, *ε*_*a*_, and *ε*_*b*_ correspond to the extinction coefficient, respectively, for the free copper(II) complex, for each addition of DNA to the copper(II) complex, and for the copper(II) complex in the fully bound form. The [DNA]/(*ε*_*a*_ − *ε*_*f*_) plot against [DNA] gave a linear relationship shown in Figure 3. The intrinsic binding constants (*K*_*b*_) for the complexes were calculated from the slope to intercept ratio. The *K*_*b*_ value for complex-**1** and complex-**2** was estimated to be 6.08 × 10^4^ M^−1^ (*R* = 0.99025 up to five points) and 5.98 × 10^4^ M^−1^ (*R* = 0.9881 up to five points) in terms of groove binding. These values are in agreement with those of well-established groove binding rather than classical intercalation [40].

Again DNA binding interaction is compared with the presence of ligand with CT-DNA. From absorption spectra, there is no change in absorption spectral band upon increasing the DNA concentration. This absorbance indicates that there is no involvement of interactions between ligand and the base pairs of DNA.

### 2.16. Ethidium Bromide Fluorescence Displacement Experiments

**Figure 4:**
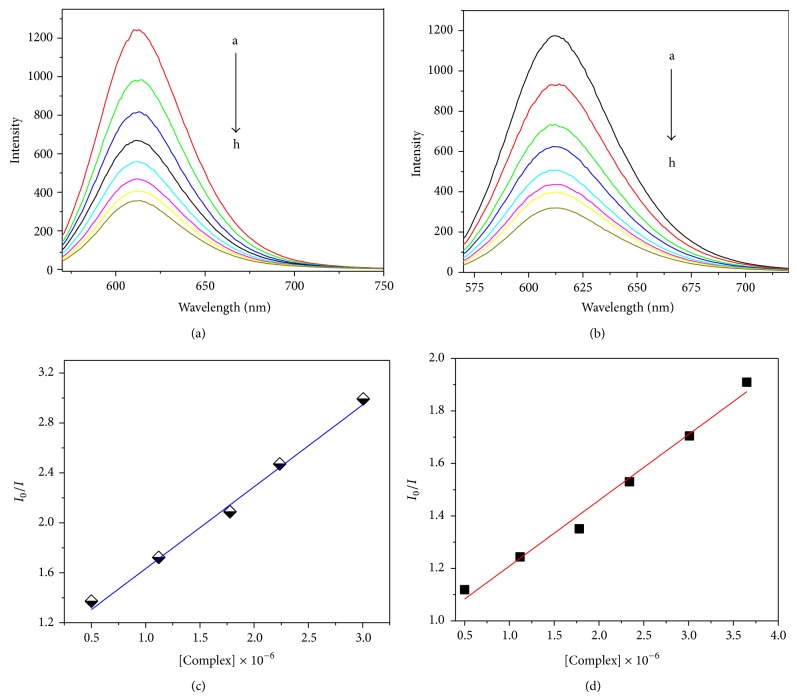
Emission spectra (a, b) of the CT-DNA-EB system in Tris-HCl buffer upon the titration of the copper(II) complex-**1** (a) and complex-**2** (b). *λ*ex = 522 nm; [EB] = 9.2 × 10^−6^ mol L^−1^, [DNA] = 1.22 × 10^−6^; [complex]:* a* 0.0,* b* 1.36 × 10^−5^,* c* 2.72 × 10^−5^,* d* 4.08 × 10^−5^,* e* 5.44 × 10^−5^, * f* 6.80 × 10^−5^,* g* 8.16 × 10^−5^,* h* 9.52 × 10^−5^ mol L^−1^. Arrow shows the intensity change upon the increase of the complex concentration. Plot of *I*_*o*_/*I* against [complex] in fluorescence quenching of CT-DNA-EB system in Tris-HCL buffer (c, d), complex-**1** (c), and complex-**2** (d), respectively.

Fluorescence quenching is a helpful method to study the reactivity of chemical and biological systems since it allows nonintrusive dimensions of substances in low concentration under physiological circumstances [41], useful information about binding mechanisms and providing clues to the nature of binding. Fluorescence intensity of a compound can be quenched as a result of molecular interactions, such as excited state reactions, molecular rearrangements, ground state complex formation, and collisional quenching. Fluorescence intensity of EB bound to CT-DNA shows a decreasing trend with the increasing concentration of the complexes as shown in Figure 4. The quenching of EB bound to DNA by the complexesis in agreement with the linear Stern-Volmer equation [42]:

(2) $$\begin{array}{c} \frac{I_{0}}{I}=1+K_{\text{sv}}\left[Q\right], \end{array}$$

where *I*_0_ and *I* represent the fluorescence intensities in the absence and presence of quencher, respectively. *K*_sv_ is a linear Stern-Volmer quenching constant and *Q* is the concentration of quencher. The *K*_sv_ value calculated from the plot is shown in Figure 4 of *I*_0_/*I* versus [complex]. The value of Stern-Volmer quenching constant (*K*_sv_) was 1.94 × 10^4^ (*R* = 0.98576 up to four points) and 1.34 × 10^4^ (*R* = 0.98818 up to four points) for complex-**1** and complex-**2**, respectively. The *K*_sv_ value in fluorescence spectral studies indicates the nonintercalative binding interaction with DNA and probable groove binding or external binding is suggested for complex-**1** and complex-**2**, which is supported by viscosity measurements. Thus the binding interaction is groove binding mode but not involved in intercalative binding. All the Stern-Volmer plots represent a good linear relationship indicating a strong affinity of the copper(II) complexes to CT-DNA.

### 2.17. Binding Parameters

When small molecules bind independently to a set of equivalent sites on a macromolecule, the binding constant (*K*_*b*_) and the numbers of binding sites (*n*) can be determined using the following equation [43]:

(3) $$\begin{array}{c} \log\left[\frac{\left(I_{0}-I\right)}{I}\right]=\log K_{b}+n\log\left[Q\right]. \end{array}$$

*K*_*b*_ and *n* are the binding constant and binding site of complex-**1 **and complex-**2 **to CT-DNA, respectively. The number of binding sites (*n*) is determined from the intercept of log⁡[(*I*_0_ − *I*)/*I*] versus log⁡[*Q*]. The number of binding sites (*n*) is 0.93 and 0.89 for complex-**1** and complex**-2**, respectively. The result indicates less association of complex-**1 **and complex-**2** to the DNA bases, also suggesting strong affinity of the complexes through surface or groove binding.

### 2.18. Cyclic Voltammetric Studies

**Figure 5:**
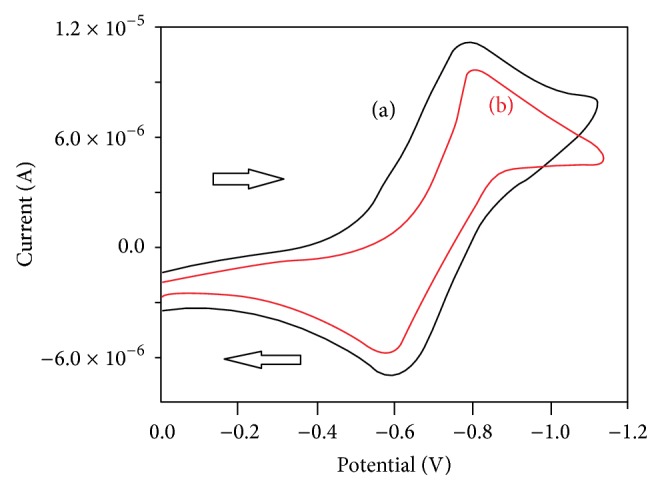
Cyclic voltammograms of complex-**1** in Tris-HCl buffer in the absence (a) and presence (b) of CT-DNA.* v* = 1 V s^−1^.

Electrochemical measurement is a most constructive technique to analyse metal-DNA interactions than spectroscopic methods [44]. The electrochemical investigations of metal-DNA interactions can provide a useful complement to spectroscopic methods, which inform about interactions with both the reduced and oxidized form of the metal. Electrochemical studies of transition-metal complexes have been extensive, and the effect of ligand concentration on potential can be used to determine formation constants. In the absence of DNA, the complexes show sharp waves peaks for both oxidation and reduction state. Upon addition of DNA both waves' peaks of *I*_pc_ and *I*_pa_ are decreased, due to large binding of copper(II) complexes to DNA and not to an increase in solution viscosity; we performed CV experiments on a mixture of copper(II) complex, which intercalates between the DNA base pairs. In this study it has been employed to recognize the nature of DNA binding of the copper(II) complexes and the result is given in Figure 5. This result indicated that interaction occurs between the CT-DNA and copper(II) complexes. The equilibrium binding constants *K*_*R*_/*K*_0_ can be calculated by using the shift value of the formal potential (Δ*E* ^0^) of Cu(II)/Cu(I) according to the Bard and Carter equation [45]:

(4) $$\begin{array}{c} \Delta E^{0}=E_{b}^{0}-E_{f}^{0}=0.0591\log\left(\frac{K_{R}}{K_{0}}\right), \end{array}$$

where *E*_*b*_^0^ and *E*_*f*_^0^ are the formal potentials of the bound and free complex forms respectively, and *K*_*R*_ and *K*_0_ are the corresponding binding constants for the binding of reduction and oxidation species to DNA, respectively. The ratio of equilibrium binding constants, *K*_*R*_/*K*_0_, is calculated to be 2.43 and 2.09 for complex-**1** and complex-**2**, respectively, which indicate the strong binding of DNA with reduced form over oxidised form of copper complexes.

### 2.19. Effect of CT-DNA on Viscosity Measurements

**Figure 6:**
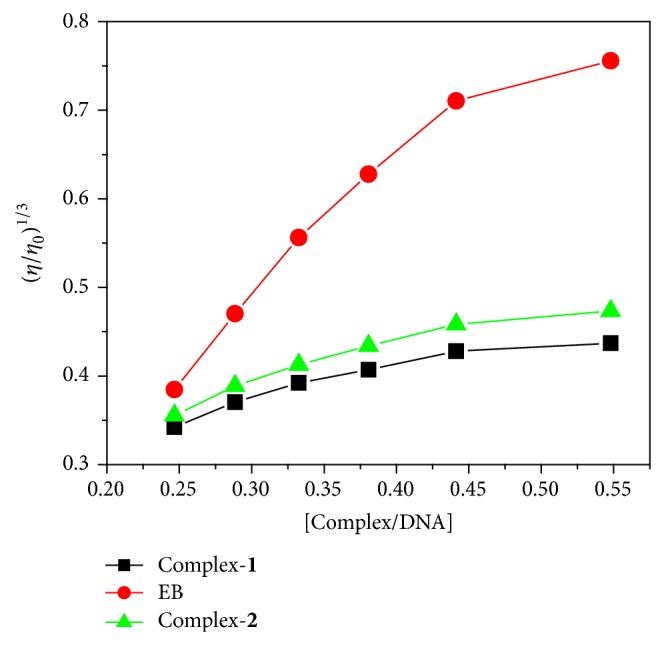
Effect of increasing amounts of copper(II) complexes (**1** and** 2**) on the relative viscosity of CT-DNA at 25°C.

Considering the nature of DNA binding of the complexes, we carried out viscosity measurements on CT-DNA by varying the concentration of added complexes. Hydrodynamic measurements, such as viscosity and sedimentation, are critical tests for a binding mode in solution in the absence of crystallographic structural data [46]. Because DNA viscosities are sensitive to the length changes of nucleic acids, a classical intercalation mode should result in lengthening the DNA helix as base pairs are separated to accommodate the binding ligand or the nonclassical intercalation could bend or kink the DNA helix, thereby decreasing its length and viscosity. From the viscosity measurements, it was observed that there is no change in the relative viscosity of the DNA solution by increasing the concentration of adding complex given in Figure 6. However, complex-**1** and complex-**2** block the intercalative interaction strongly and hence the negligible changes in the relative DNA viscosity observed. This is in conformity with its lowest DNA binding affinity. Similarly, the presence of hydroxyl group on the naphthyl ring would also sterically hinder the partial insertion of the ligand ring in between the DNA base pairs, leading to no change in relative viscosity of DNA. This suggests that these complexes interact with CT-DNA through groove binding mode.

### 2.20. Fluorescence Microscopy Study of Cu-DNA Complexes

**Scheme 2:**
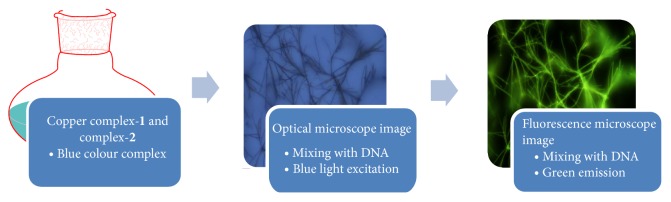
Synthetic route of fluorescent green microwires of complex-**1** and complex-**2** with DNA.

**Figure 7:**
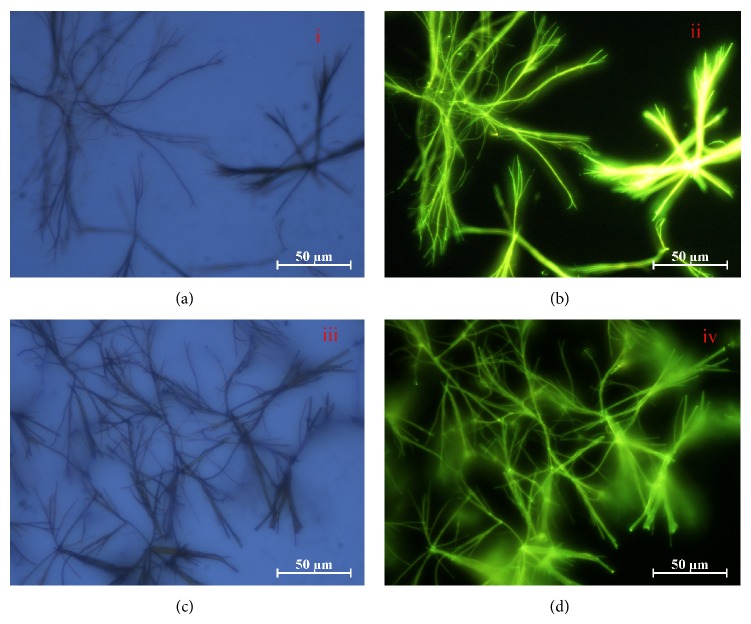
Optical and fluorescence micrographs of complex-**1** (a and b) and complex-**2** (c and d) with DNA microwires, respectively. The fluorescence micrograph was obtained by the excitation of the sample with blue light between 450 and 490 nm.

The fluorescence micrograph showed formation of microwires shaped copper complex with DNA (Figure 7). The obtained green microwires were characterized by using fluorescence microscopy to investigate their optical waveguide properties. The fluorescence micrograph was obtained by the excitation of the sample with blue light between 450 and 490 nm (Scheme 2). This investigation clearly demonstrates that Cu complexes (**1** and** 2**) molecules have been incorporated into the DNA microparticles and arbitrarily distributed in the nanoparticles. This leads to an impressive increase in the fluorescence intensity of the polymer even on high dilution with DNA. During the formation of the doped microparticles, hydrophobic and *π*-*π* interactions induce the aggregation of DNA and Cu complexes (**1** and** 2**) molecules into microparticles [47]. This clarification suggested that the microwires absorbed the excitation light and propagated the fluorescence emission toward the tips, thereby exhibiting strong wave guiding behaviour. The DNA-Cu complexes microwires show significant optical waveguide properties due to the strong fluorescence emission.

### 2.21. Antibacterial Activity

**Figure 8:**
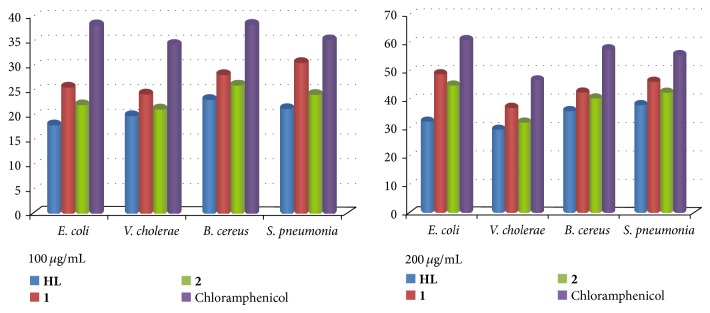
Comparison of the antibacterial activity of** HL**, complex-**1**, and complex-**2** with standard chloramphenicol drug. DMF solvent does not show any activity.

Antibacterial activity of the ligand and its copper(II) complexes is tabulated in Figure 8. The biological activity of the synthesized ligand and its compounds are compared with standard antibiotic chloramphenicol drug. From this study it is inferred that all complexes have higher activity than ligand but lower than antibiotic. Here height of the bar represents the activity of complexes and ligand with respect to standard antibiotic. The increased activity of the metal chelates can be explained by overtone concept and the Tweedy chelation theory [48]. The variation in the activity of copper(II) complexes against some different organisms depends on either the impermeability of the cells of the microbes [49] or difference in ribosome of microbial cells and also activity increases with increasing the concentration of complexes. In a complex, polarity of metal ions reduces due to partial sharing of its positive charge with donor groups of ligand and delocalization of *π*-electron into the whole chelate ring. Lipids and polysaccharides are important constituents of cell walls and membranes, which are preferred for metal ion interaction. This increased lipophilicity also helps the penetration of the bacterial cell membranes and restricts further growth of the microorganisms. Due to higher lipophilicity, complex-**1 **and complex-**2 **exhibit higher antibacterial activity than free ligand.

### 2.22. Antioxidant Activity

We investigated the free radical scavenging ability of the newly synthesized ligand and its complexes using DPPH. The DPPH radical is one of the most commonly used substrates for fast evaluation of antioxidant activity because of its stability and simplicity of the assay. In DPPH assay, the ligand and its complexes act as donors of hydrogen atoms or electrons in transformation of DPPH radical into its reduced form DPPH-H. The breaking of the OH bond is considered to be one of the most important physicochemical parameters involved in the definition of the antioxidant potency of phenolic derivatives [50]. Phenols are also excellent chain-breaking antioxidants and good ^1^O_2_ quenchers [51]. Lower absorbance values of the reaction mixture indicated higher free radical scavenging activity. The DPPH radical scavenging ability of the ligands and their complexes is shown in Table 2. It is inferred that free radical scavenging activities of synthesized compounds are concentration dependent and the activity of complexes increases with increasing its concentration. Ascorbic acid, a phenolic antioxidant, used as a standard, showed stronger antioxidant activity than that of synthesized compounds. It can be concluded that complexes are more scavenging activity than ligand due to partial sharing of positive charge with hole organic moiety and also electron releasing hydroxyl and methyl group present in the ligand moiety. After the complexation with metal ions reveals that the antioxidant activity increases due to the presence of positively charged meal ions as well as electron donating groups present in the moiety, so complexes have a strong potential to be applied as scavengers to eliminate radicals [52].

## 3. Conclusion

Synthesis and characterization of two mononuclear copper(II) complexes of N_2_O donor set have been performed. All complexes are pentacoordinated formulated as [Cu(L)(Cl)(H_2_O)] (**1**) and [Cu(L)(Br)(H_2_O)] (**2**). The electrochemical study of these complexes showed a quasi-reversible one-electron transfer process. DNA binding properties of the copper(II) complexes with DNA have been investigated by absorption spectra, fluorescence spectra, and voltammetry measurements. All results indicate that the copper(II) complexes bind to CT-DNA via groove binding mode. Complexation between the copper complexes (**1** and** 2**) and the anionic DNA molecules appears to stiffen the backbones of the former leading to a green color in its fluorescence emission. The observed enhancement of fluorescence may be utilized in sensing DNA. DFT calculations are used to observe the electronic structure and the electronic properties of copper(II) complexes. Furthermore,* in vitro* antioxidant activity of copper(II) complexes also exhibits the effective scavenging of DPPH radicals. In addition, the result of antibacterial studies confirmed that ligand and complexes are bioactive showing good antimicrobial property. It has also been proposed that concentration plays a vital role in increasing the degree of inhibition; as the concentration increases, the activity increases.

## Supplementary Material

Here we show the IR, ^1^H-NMR and ESI-mass spectra of ligand (HL). Also we have added the IR and mass spectra of complexes 1 & 2. These spectral data confirm the formation of ligand and complexes 1 & 2.

## Figures and Tables

**Table 1:** UV-Vis spectral and electrochemical data of complex-**1** and complex-**2**.

Compound	UV-Vis data *λ*, nm (*ε*, dm^3^ mol^−1^ cm^−1^)^a^	Electrochemical data^a^		
		*E*_pc_ (V)	*E*_pa_ (V)	*E*_1/2_ (V)
**1**	262 (10,532), 319 (8,037), 365 (3,125), 667 (236)	−0.779	−0.602	−0.690
**2**	254 (9.753), 327 (6,534), 362 (2,176), 672 (154)	−0.712	−0.597	−0.654

^a^In DMF; electrochemical data recorded in mV, at 298 K and scan rate 100 mVs^−1^; *E*_1/2_ = (*E*_pc_ + *E*_pa_)/2.

**Table 2:** Antioxidant activity (*μ*M) of ligand and metal complexes (**1** and **2**) at different concentrations using DPPH assay.

Compounds	Concentration (*μ*g/mL)						
	0	25	50	75	100	125	150
**AA**	0	9.98	20.24	29.64	40.84	47.23	54.57
**HL**	0	7.57	13.54	20.65	26.87	32.98	36.43
**Complex-1**	0	8.52	15.87	25.76	34.82	41.74	47.32
**Complex-2**	0	8.12	16.23	23.76	33.67	39.12	43.18
