# Supplementary material for: Synthesis and Characterisation of Copper(II) Complexes with Tridentate NNO Functionalized Ligand: Density Function Theory Study, DNA Binding Mechanism, Optical Properties, and Biological Application
Source: Bioinorg Chem Appl. 2014 Oct 16;2014:104046. doi: 10.1155/2014/104046 (PMC4214117; doi:10.1155/2014/104046)
Supplement: Supplementary file 1 — Here we show the IR, 1H-NMR and ESI-mass spectra of ligand (HL). Also we have added the IR and mass spectra of complexes 1 & 2. These spectral data confirm the formation of ligand and complexes 1 & 2. [file 104046.f1.doc]

**Supporting Information for:**

**Synthesis and characterisation of Copper (II) complexes with tridentate NNO functionalized ligand: DFT study, DNA binding mechanism, optical properties and biological application**

Madhumita Hazra^1,3^, Tanushree Dolai^1^, Akhil Pandey^2^, Subrata Kumar Dey^3*^, Animesh Patra^1*^

*^1^Postgraduate Department of Chemistry, Midnapore College, Midnapore- 721101, India ^2^Department of Microbiology, Midnapore college, Midnapore-721101, India,*

*^3^Depatment of Chemistry, Sidho-Kanho-Birsha University, Purulia, West Bengal, India*

**Figures’ legend**

**Fig. S1** IR spectra of **HL** and Complex-**1.**

**Fig. S2** The ^1^H- NMR spectrum of the ligand (**HL**).

**Fig. S3** The ^13^C-NMR spectrum of the Ligand (**HL**).

**Fig. S4** ESI-mass spectra of complex-**1**.

**Fig. S5** ESI-mass spectra of complex-**2**.


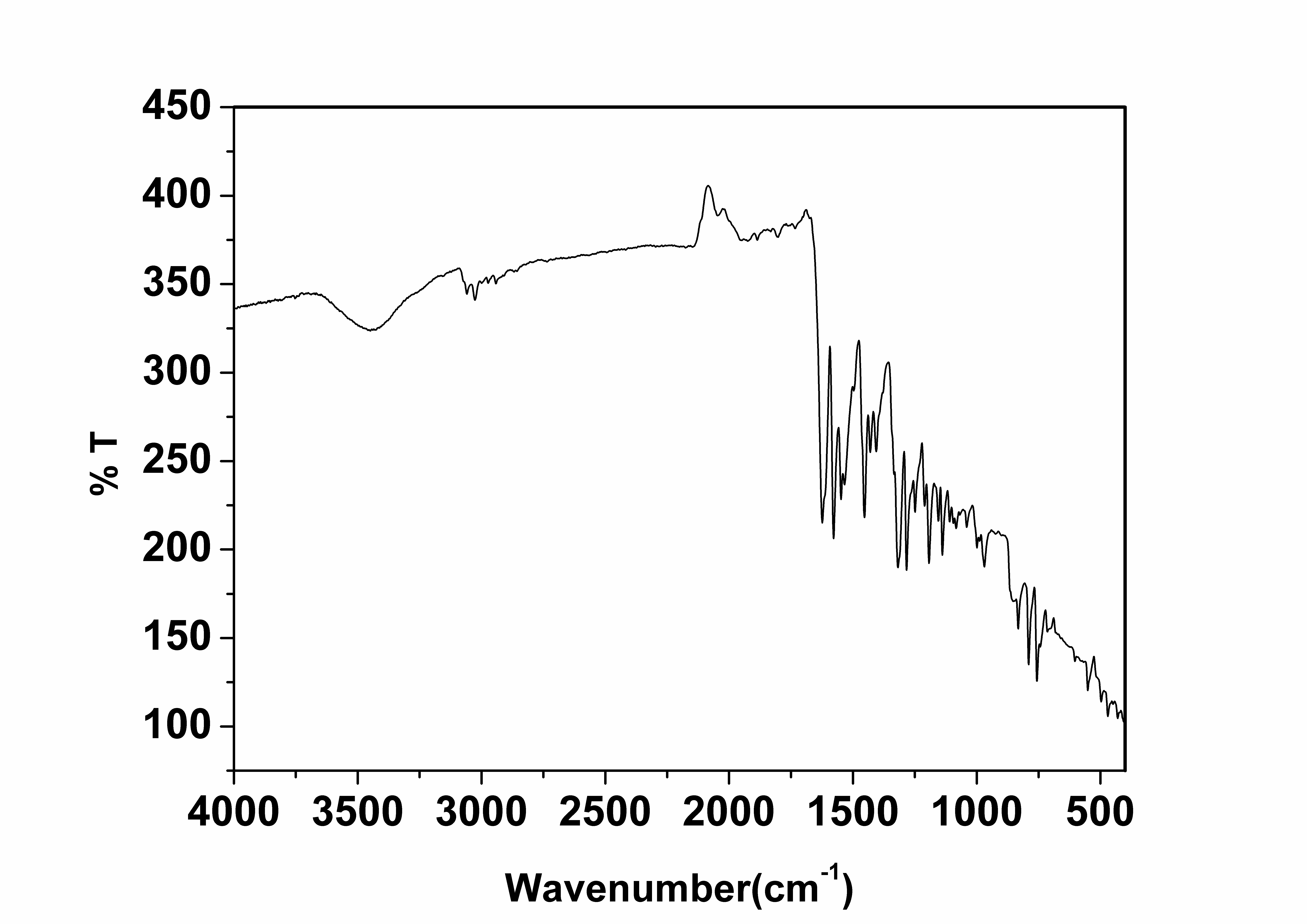

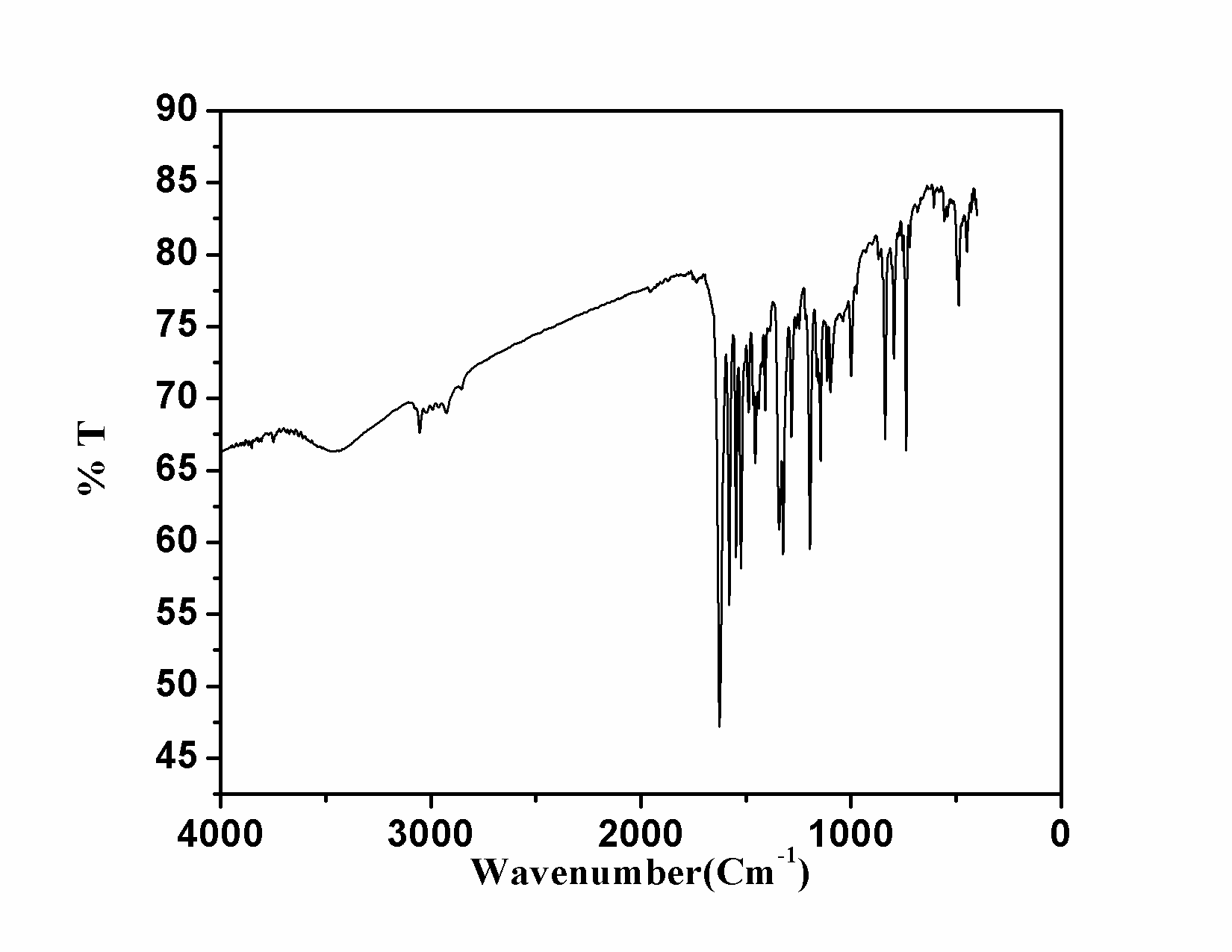


**Fig. S1** IR spectra of **HL** and Complex-**1.**

**
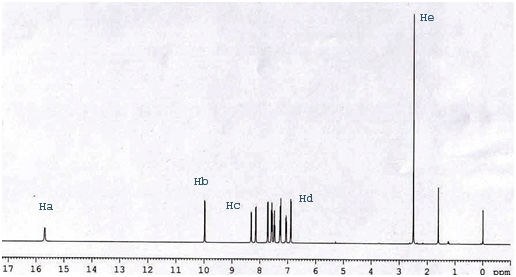
**

**Fig. S2** The ^1^H- NMR spectrum of the ligand (**HL**).


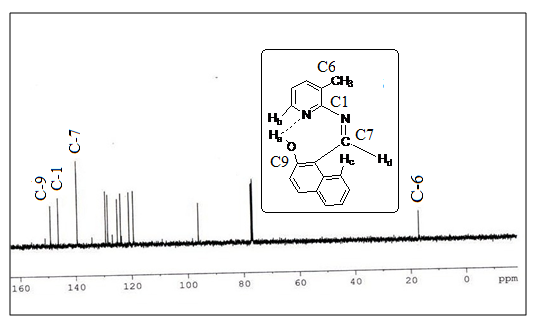


**Fig. S3** The ^13^C-NMR spectrum of the Ligand (**HL**).

**
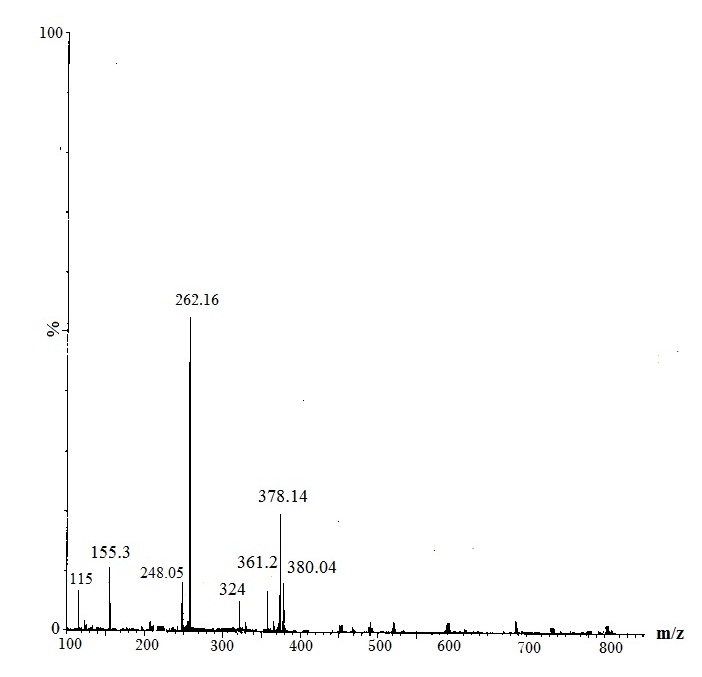
**

**Fig. S4** ESI-mass spectra of complex-**1**


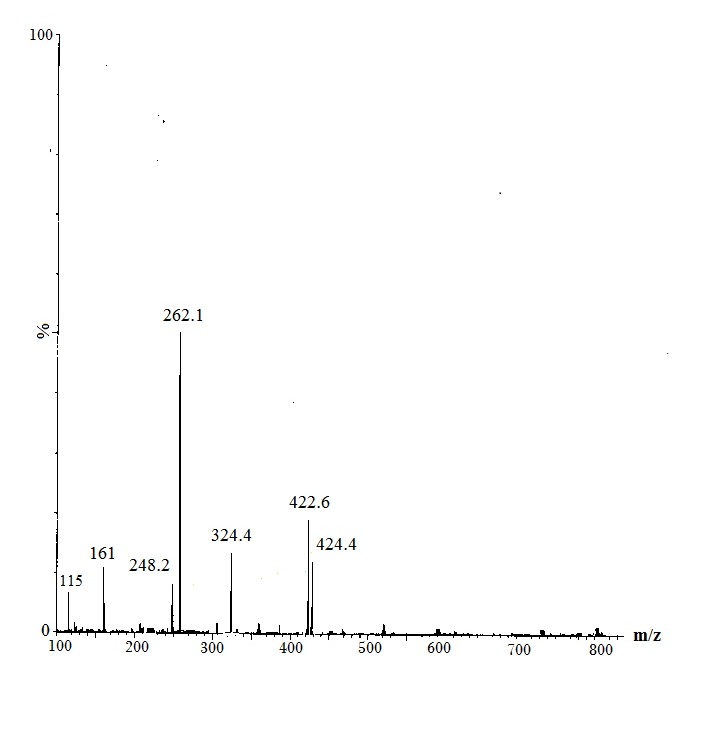


**Fig. S5** ESI-mass spectra of complex-**2**

**Table S1**

Selected geometrical parameters for **1** and **2** (distance in Å and angle in degrees)

| Bond distance in Å | | |
| --- | --- | --- |
|  | **1** | **2** |
| N1-C1 | 1.3448 | 1.3693 |
| N2-C2 | 1.4224 | 1.4185 |
| N2-C7 | 1.3100 | 1.3200 |
| Cu-N1 | 1.2900 | 1.2820 |
| Cu-N2 | 1.7071 | 1.6996 |
| Cu-O1 | 1.3274 | 1.3241 |
| Cu-O2 | 1.7500 | 1.7500 |
| Cu-Cl | 2.1600 | - |
| Cu-Br | - | 2.2925 |

| Bond angle in degrees(^0^) | | |
| --- | --- | --- |
| **1** **2** | | |
| N1-C1-N2 | 75.20 | 74.70 |
| N1-Cu-N2 | 67.14 | 67.46 |
| N1-Cu-O1 | 143.55 | 137.79 |
| N2-Cu-O1 | 102.55 | 98.15 |
| N1-Cu-O2 | 90.41 | 89.93 |
| N2-Cu-O2 | 144.18 | 144.17 |
| N1-Cu-Cl | 151.72 | - |
| N2-Cu-Cl | 139.33 | - |
| N1-Cu-Br | - | 149.28 |
| N2-Cu-Br | - | 140.41 |

* O2 is the oxygen atom of H_2_O molecule.
